# High sleep quality can increase the performance of CrossFit® athletes in highly technical- and cognitive-demanding categories

**DOI:** 10.1186/s13102-021-00365-2

**Published:** 2021-10-28

**Authors:** Kristina Klier, Selina Dörr, Annette Schmidt

**Affiliations:** 1 Fakultät Für Humanwissenschaften, Institut Für Sportwissenschaft, Universität der Bundeswehr München, Werner-Heisenberg-Weg 39, 85577 Neubiberg, Germany; 2 Forschungs- und Lehrgebiet Gesundheit, Betriebliche Gesundheitsförderung und Prävention, Universität der Bundeswehr München, Werner-Heisenberg-Weg 39, 85577 Neubiberg, Germany; 3 Forschungs- und Lehrgebiet Sportbiologie, Universität der Bundeswehr München, Werner-Heisenberg-Weg 39, 85577 Neubiberg, Germany

**Keywords:** Athletes, Fitness, Health, Performance, Sleep quality

## Abstract

**Background:**

In current sports science, the important role of sleep quality for health and peak performance is well acknowledged. More precisely, it is evident that the negative combination of stressful factors, low resources, and bad sleep habits causes short-term performance losses as well as long-term health consequences. As the maximization of human performance consisting of multiple fixed workloads is the main motivation in CrossFit® (CF), the aim of the present study was to investigate the influence of high sleep quality on performance in CrossFit® athletes (CFA) and the different training categories that are addressed in CF.

**Methods:**

In total, 149 CFA (81 females, 68 males; 32.01 ± 7.49 years old, 2.56 ± 1.77 active years in CF) filled in the online survey comprising the Pittsburgh Sleep Quality Index (PSQI) and CF performance-specific questions.

**Results:**

It was found that CFA with high sleep quality reported higher values in all performance-related outcomes. Especially in Hero-/Girl-Workouts (χ2 = (1, n = 83) = 9.92, *p* = .002, φ = 0.37) and Gymnastics (χ2 = (1, n = 129) = 8.74, *p* = .003, φ = 0.28), performance differed significantly between good and poor sleeping CFA. Since those CF categories are highly technical- and cognitive-demanding high sleep quality seems to play a fundamental role in complex motor skill learning and performance development.

**Conclusions:**

These results reveal for the first time the positive effects of high sleep quality for performance in CFA and could be used as basis for future studies. Future research should also develop and empirically test suitable interventions to foster high sleep quality in CFA.

*Trial Registration*: The study was conducted according to the guidelines of the Declaration of Helsinki, and approved by the Ethics Committee of University of the Federal Armed Forces Munich, Germany (06/04/2018).

Within the last decades, sleep is focused in various research fields because of its essential functions for human well-being [16]. Besides psychophysiological recreation, it is of particular importance for learning and memory consolidation [17]. Moreover, in terms of health and performance, it is discussed as enhancing resource and at the same time as determining factor [6, 19]. Likewise, growing number of researches has dealt with the relationship between sleep and physical activity [8]. Whereas regular physical activity seems to promote work-life-balance and even fosters high sleep quality, especially among athletes, an accumulation of sleep problems can be identified [26]. Further, the direction of this relationship remains open: No precise statements can be made about whether it is the level of activity that affects sleep, or whether it is the quality or duration of sleep per se that affect sports performance [27, 41].

However, various studies have shown the importance of high sleep quality and its positive effects on the circadian rhythm as well as on the hormonal and immune system while sleep disturbances negatively affect mood and cause a decline in fine motor skills, maximum strength, and aerobic performance [14, 24, 36, 40]. Furthermore, high sleep quality is associated with high motivation, attention and concentration, and decreases in perceived exhaustion and pain perception [12, 35]. Accordingly, Chandrasekaran and colleagues [6] recommend to keep cognitive, emotional and environment-related influences as low as possible in order to be able to fall asleep timely and sleep uninterruptedly, and consequently, to be more concentrated and more attentive during waking phases. In this context, body temperature and light conditions (i.e., brightness) seem the easiest factors to influence, followed by noise, excitement/tension and fear. Especially before competitions, high arousal and fearful thoughts seem to appear more often. For example, Lastella and colleagues [25] have shown a variance of 4–5% in mood and general well-being after bad night sleep. In line, van Ryswyk et al. [34] have demonstrated that a tailored sleep optimization program led to an improvement in general well-being in male football players. Bonnar et al. [1] have proved that sleep optimization tailored to the individual athlete (e.g., sleep extension, power naps, sleep hygiene) significantly increases sleep quality and well-being. Hence, Kellmann and colleagues [21] have summarized the risk of short-term performance losses up to long-term health consequences due to the negative combination of stressful factors, low resources, and bad sleep habits in terms of sleep quality and sleep duration.

In general, the majority of the studies has focused on the circumstances of the duration respectively the quality of sleep, or the factors causing sleep deprivation and its consequences. Mostly, the examined sports setting has been localized in cycling, swimming, rowing, basketball and football as well as in regard to the night(s) before and after competition(s). In particular, the state of research in the training setting as well as in concerns of sleep interventions remains insufficiently considered. However, there is broad consensus on the need for sleep monitoring and documentation [1, 10, 33]. There are currently no explicit recommendations or guidelines on sleep hygiene or sleep monitoring [18, 30]. While on the one hand, research groups consider the potentials and limitations of current sleep applications and measurement methods [20, 38], on the other hand, drafts of formulations for sleep and behavioral rules are continually emerging [23, 31, 37].

Accordingly, the sporting paradigm seems to shift from “faster, higher, stronger” to “the best better rest”. This approach of sleeping to perform might also be promising in the fitness setting. For instance, CF is becoming most popular within the last years. CF was established in 2000 by Greg Glassman as a trend sport, now being even more a fitness culture itself. It is characterized by multiple workouts with fixed training loads aiming for general physical preparedness, i.e., maximize full body performance to be prepared for the unknown and unexpected [15]. The highly intense sport combines various categories like exercises of Olympic Weightlifting and gymnastics with self-weight exercises and cardiovascular endurance exercises such as rowing and running. Due to the constantly varied functionality, all performance-related aspects consisting endurance, strength, flexibility, speed, balance and coordination are systematically optimized. Accordingly, the main goal of CF is to develop healthy and powerful bodies, and to achieve highest possible functional fitness level [11]. A CF training session usually lasts one hour and consists of beforehand set daily tasks including warm-up, skill training (skill development), high-intensity training (conditioning, known as WOD (Workout Of the Day)) and stretching. During training each CFA chooses load, intensity, set times etc. adapted to his/her individual performance level. Despite these individual load norms, the workouts are completed by all in-site CFA at the same time in order to increase extrinsic and intrinsic motivation and will power. Thus, the nature of CF is very competitive. To compare the increase in performance as well as to track the personal performance development, WODs are standardized, and at irregular intervals but always under the same conditions so-called benchmark workouts (known as Hero- (often particularly long and hard)/Girl-WODs (mostly short and intense)) should be repeated. Besides its competitive nature, CF maintains its own philosophy, which, in addition to the training principle described, also promotes other influencing factors such as an active lifestyle and healthy diet.

As mentioned above, in spite of the growing research interest, there is still a lack and an unequivocal evidence respectively in terms of sleep, health and performance [6, 39]. According to Kirschen et al. [22], who reviewed 19 studies comprising a total of 12 different sports, especially sports with high technical, tactical and speed requirements seem more prone to the negative effects of sleep deprivation. This, in turn, is of particular interest for the technical and speed-demanding aspects of CF. Therefore, the aim of the present study was to investigate for the first time the influence of high sleep quality on performance in CFA and the different categories of CF training. Based on the described body of research, we expect the performance of CFA developing respectively increasing more positively in addition to high sleep quality than compared to persistently poor sleep.

## Methods

### Participants

In total, 178 participants clicked the online survey. Due to incomplete answers, 20 participants were excluded as well as 9 others who did not met the inclusion criteria. To be included, participants needed to actively practice CF and do not consume caffeinated drinks immediately before training. Latter exclusion criterion was set to provide the established negative effects of caffeine on sleep quality and performance [9]. In general, participation was voluntary and without consideration.

### Study procedure

The study was conducted using a common online survey tool that met the university’s ethics and privacy policy, and was in accordance to the guidelines of the Declaration of Helsinki. For investigation, we developed a questionnaire based on standardized scales and the current state of the literature, and shared the link on local CF platforms and social media. Finally, the questionnaire was online available for 33 days.

### Materials

#### Pittsburgh Sleep Quality Index

To gather all relevant information about participants’ sleep we used the standardized Pittsburgh Sleep Quality Index (PSQI; [5]. It is a reliable and valid instrument (total score of Cronbach’s α = 0.80, test–retest reliability r = 0.82 to 0.89) to collect and categorize individual sleep quality. The questionnaire comprises 19 items for self-assessment of one's own sleep behavior within the last four weeks. The last question (question about partner and/or roommate) will not be included in the quantitative evaluation. From the remaining 18 questions seven individual components can be derived: *(1)* subjective sleep quality (one item), *(2)* sleep latency (two items), *(3)* sleep duration (one item), *(4)* sleep efficiency (three items), *(5)* potential sleep disorders (nine items), *(6)* sleep medication consumption (one item), and *(7)* daytime sleepiness (two items). The value of the individual components ranges between zero and three. The index value itself ultimately represents the sum of the seven individual component values and can assume values between a minimum of zero and a maximum of 21. In general, values < 5 refer to high sleep quality, 6–10 poor sleep quality, and > 10 chronic sleep disturbances [5]. Reference values exist both for samples with and without diagnosed sleep problems.

Related Questions about Influence On Mental And Physical Performance We extended the sleep questioning part by few further questions. First, participants should answer whether (yes/no) respectively which method(s) (e.g., meditation, autogenic training, avoidance of blue light before going to bed) they use to improve their sleep quality. For the latter question, participants could choose multiple answers as well as “nothing” or specify a method that is not listed under "other" in the free text field. Finally, they were asked in two separate items to rate on a four-point Likert scale the role of their individual sleep behavior for their physical and mental performance.

#### CF-specific questions

The questions regarding the CF performance development were oriented to the basic CF disciplines, as these are exercises that are performed regularly. To determine a positive or negative development of the performance, all CF-specific questions were related to the training within the last four weeks. Thus, a reliable statement on changes could be made, and at the same time, all performance components inquired. In total, we formulated six items for this CF-specific questioning part, in which participants were asked about the state or improvement of their performance, which they could either affirm or deny on a nominal scale. Additionally, we added the option “I cannot provide any information” in case the questioned exercise has not been carried out within the last four weeks. First, the development of the general fitness level should be rated, followed by the maximum strength (1 RM, i.e., maximum weight, which can be mastered one time) and strength endurance (5 RM, i.e., maximum weight, which can be mastered five times) of the basic exercises of weightlifting: deadlift, squat, bench press, snatch, reposition, push. Further, participants were asked to report their performance development of so-called Hero-WODs and Girl-WODs. These are standardized workouts with fixed repetitions and weights that are ideal for comparing performance. Likewise, there are two different variants to improve workout performance: On the one hand, the time to complete the given exercises and repetitions can be shortened (time improvement), and on the other hand, the workout can be completed with a higher weight or more difficult form of exercise (implementation improvement). Both variants counted for this item equivalent as an improvement and were not differentiated. Finally, the progress in gymnastic exercises such as handstand, pull-up, dips or muscle-up, and cardiorespiratory demands during rowing, swimming, running, or cycling was inquired.

The whole survey ended with demographic (age, gender, type of employment) and anthropometric (height, body weight) data. At last, participants should specify how long they have been actively practicing CF in order to be able to better evaluate and classify possible side effects (e.g., steeper learning curve for beginners, more forced training planning for advanced CFA).

### Statistical analysis

The statistical analysis was carried out using the data processing programs Excel (Microsoft, 2019) and SPSS Statistics Version 27 (IBM, Inc., Chicago, IL, 2021). The level of significance was set a priori at α < 0.05 [2]. According to PSQI we grouped participants as good or poor sleepers. CF performance was analyzed descriptively. Further, as it was two independent samples with a nominal scale level, we calculated chi-square and Phi coefficient in order to test existing relationship between sleep quality and CF performance. Only the results of participants who have decided for or against an improvement in performance were included. All data sets with the selection “I cannot provide any information” were excluded from the respective analysis.

## Results

### Descriptive Statistics

After excluding the total of 29 invalid questionnaires, 149 valid data records have been included into the statistical analysis. Of these, 81 participants were female and 68 were male. Their age ranged from 18 to 60 years, and their active CF training experience ranged from few months up to ten years. Further descriptive characteristics of participants are shown in Table 1.

**Table 1:** Descriptive characteristics of participants (x̅ ± SD)

	Total (N = 149)	Male (n = 68)	Female (n = 81)
Age (years)	32.01 ± 7.49	32.07 ± 7.48	31.95 ± 7.53
Height (cm)	173.93 ± 8.99	180.65 ± 6.39	168.30 ± 6.69
Weight (kg)	74.92 ± 12.51	83.40 ± 9.28	67.72 ± 10.19
BMI (kg/m^2^)	24.65 ± 3.08	25.55 ± 2.47	23.89 ± 3.35
CF experience (years)	2.56 ± 1.77	2.50 ± 1.66	2.61 ± 1.87
Main training time(s)	Afternoon (28%); Evening (48%)	Afternoon (25%); Evening (51%)	Afternoon (31%); Evening (44%)

[BMI = Body Mass Index; Afternoon = 3 to 6 pm; Evening = 7 to 10 pm]

In terms of sleep behavior, participants reported 7.08 h of total sleep duration (± 0.98 h). 87% of all participants (n = 129) experienced various sleep interruptions up to once a week (e.g., nocturnal awakening due to feelings of cold/heat, urination, breathing difficulties, nightmares). In addition, only 22% (n = 33) rated their sleep quality within the last four weeks as “good”, 58% (n = 86) as “rather good”, and 20% (n = 30) as “rather or very bad”. There were particularly great differences in the duration of falling sleep. While the average time was 19.89 min (± 17.28 min), the maximum sleep latency was 90 min. Likewise, the reported daytime sleepiness seemed rather bad with 74 participants (50%) struggling once a week and 36 participants (24%) up to twice a week with staying awake and tackling the momentum during everyday activities.

Referring to the complete analysis of PSQI, 93 participants (62%; 46 males, 47 females) could be assigned as *good sleepers* (GS) and 56 participants (38%; 22 males, 34 females) as *poor sleepers* (PS).

### Performance development

**Fig. 1:**
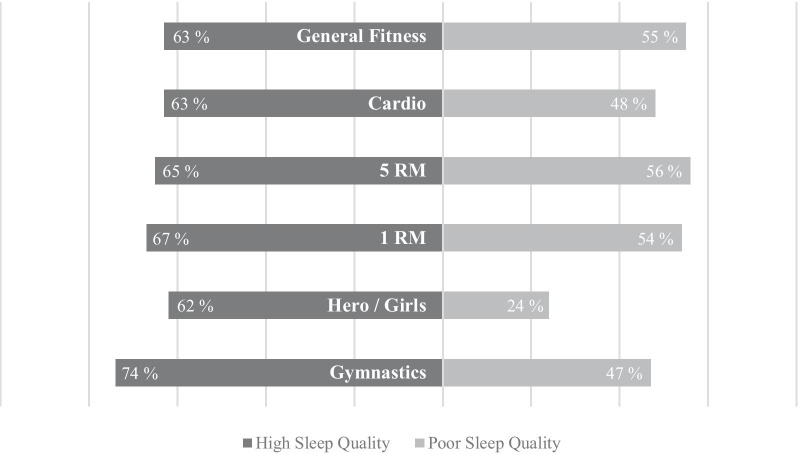
Percentage of increased performance within the last four weeks in comparison of good to poor sleeping CFA

To analyze the influence of high sleep quality on performance development of CFA we calculated the difference of the named CF categories by facing the answers of GS with PS. Expressed in percentage, overall, more subjects of GS reported a performance improvement than PS. Although this general difference is not significant, it could be found in all CF-specific outcomes (see Fig. 1).

In all analyzing aspects, no difference could be found between males and females. General survey results are shown in Tables 2 and 3 (* indicates significant differences between good and poor sleeping CFA (p < 0.05)).

**Table 2:** General overview of results referring CF performance

Variable			High sleep quality		Poor sleep quality
General Fitness	Absolute (Relative) Frequency	Improvement	59 (63%)		31 (55%)
		No Improvement	18 (19%)		13 (23%)
		Not Applicable	16 (17%)		12 (21%)
				χ^2^ = (2, N = 149) = 0.96, p = .62, φ = 0.08	
Cardio	Absolute (Relative) Frequency	Improvement	52 (63%)		21 (48%)
		No Improvement	30 (37%)		23 (52%)
			χ^2^ = (1, n = 126) = 2.28, p = .13, φ = 0.15		
5 RM	Absolute (Relative) Frequency	Improvement	42 (65%)		24 (56%)
		No Improvement	23 (35%)		19 (44%)
			χ^2^ = (1, n = 108) = 0.52, p = .47, φ = 0.09		
1 RM	Absolute (Relative) Frequency	Improvement	49 (67%)		23 (54%)
		No Improvement	24 (33%)		20 (47%)
			χ^2^ = (1, n = 116) = 1.60, p = .21, φ = 0.14		
Hero-/Girl-WODs	Absolute (Relative) Frequency	Improvement	31 (62%)		8 (24%)
		No Improvement	19 (38%)		25 (76%)
			χ^2^ = (1, n = 83) = 9.92, p = .002 *, φ = 0.37		
Gymnastics	Absolute (Relative) Frequency	Improvement	61 (74%)		22 (47%)
		No Improvement	21 (26%)		25 (53%)
			χ^2^ = (1, n = 129) = 8.74, p = .003 *, φ = 0.28

**Table 3:** General overview of results referring other factors

Variable			High sleep quality	Poor sleep quality
Sleep quality	Absolute (Relative) Frequency	Very Good	33 (35%)	0 (0%)
		Rather Good	59 (63%)	27 (48%)
		Rather Bad	0 (0%)	27 (48%)
		Very Bad	1 (1%)	2 (4%)
Sleep duration	Absolute (Relative) Frequency	3–4 Hours	0 (0%)	1 (2%)
		5–6 Hours	7 (8%)	33 (59%)
		7–8 Hours	82 (88%)	20 (36%)
		9–10 Hours	4 (4%)	2 (4%)
Influence on mental performance	Absolute (Relative) Frequency	Small–No Influence	10 (11%)	6 (11%)
		Moderate–strong influence	83 (89%)	50 (89%)
Influence on physical performance	Absolute (Relative) Frequency	Small–no influence	11 (12%)	11 (20%)
		Moderate–strong influence	82 (88%)	45 (80%)
Sleep tracking/optimization	Absolute (Relative) Frequency	Yes	32 (34%)	27 (48%)
		No	61 (66%)	29 (52%)

#### General fitness level

The first CF performance item asked about participants’ general fitness level. GS and PS values were according to chi-square test with χ2 = (2, N = 149) = 0.96, *p* = 0.62, φ = 0.08 not significant.

#### Cardio

Regarding cardiorespiratory outcome, again, although a difference could be seen between the two groups, it is with χ2 = (1, n = 126) = 2.28, *p* = 0.13, φ = 0.15 not significant.

#### 5 RM

The analysis of the 5 RM also showed different results, but statistically, the difference with χ2 = (1, n = 108) = 0.52, *p* = 0.47, φ = 0.09 is not significant.

#### 1 RM

Results were similar when looking at the performance development under the aspect of maximum strength. Expressed in percentage, the difference is after continuity correction with χ2 = (1, n = 116) = 1.60, p = 0.21, φ = 0.14 not significant.

#### Hero-/Girl-WODs

The analysis of the standardized Hero-/Girl-WODs revealed different results. Thereby, not only the comparison of the percentage values is noticeable, but also the results of the statistical test procedure. With χ2 = (1, n = 83) = 9.92, p = 0.002, φ = 0.37 this found difference is statistically significant.

#### Gymnastics

When looking at the gymnastics elements, another significant difference could be found (χ2 = (1, n = 129) = 8.74, p = 0.003, φ = 0.28).

### Other factors

#### Sleep quality and duration

Even in terms of the subjective assessment of one's own sleep quality as well as in the analysis of sleep duration, we found a connection to group assignment: Answers of PS tend to be more negative than of GS.

#### Influence on mental and physical performance

**Fig. 2:**
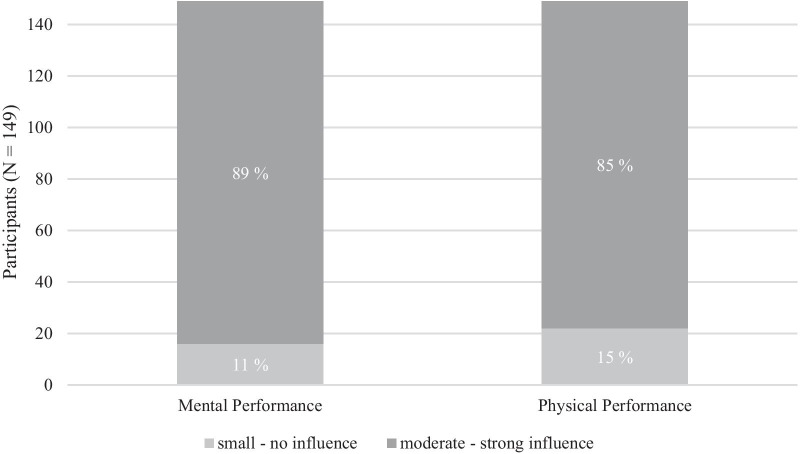
Rating of influence of high sleep quality on mental and physical performance

In a further item, participants were asked to assess the influence of their subjective sleep quality on their performance, both mentally and physically. Interestingly, regarding the whole sample, the influence of high sleep quality on mental performance was rated four percent higher than that of physical performance (see Fig. 2).

In addition to the question of how important participants rated sleep quality, they were asked whether they use any methods to optimize their sleep quality. Of the 149 participants, two thirds (60%, n = 90) answered not to intentionally assess and optimize their sleep. The others reported, for example, to avoid bright light in the evening (26%, n = 39), meditate (9%, n = 13), read a book (3%, n = 4), listen to music (19%, n = 28), or do some breathing or stretching exercises (4%, n = 6) to establish a healthy sleep hygiene.

## Discussion

The aim of the present study was to investigate the influence of high sleep quality on performance-specific outcomes in CFA differentiated on the different categories of CF training. We hypothesized that there would be a significant difference in the performance development of CFA with good sleep behavior, i.e., high sleep quality, compared to those with poor sleep. Sleep is referred to as good or healthy sleep when it comprises sufficient duration (recommended 7–9 h), efficiency (≥ 85%), depth (15–20% deep and REM sleep, 55–60% light sleep), low sleep latency (≤ 15 min) and one or fewer interruptions [28]. If these characteristics are partially or completely absent, sleep is considered poor or unhealthy. Thus, whereas the performance development is likely to decrease due to persistently poor sleep, CF performance will develop more positively in addition to high sleep quality. As this is the first study considering the influence of sleep in CF, our findings, i.e., calculated percentage values, are consistent with current literature revealing that high sleep quality can lead to an increased sports performance.

### Difference in CF performance development

Although we could not accept our hypothesis in total due to partially not significant results, we found differences between good and poor sleeping CFA in all CF categories. In particular, Hero-/Girl-WODs and Gymnastics were found to be statistically significant.

As mentioned, CFA use Hero-/Girl-WODs to assess their own performance improvements over time as well as to compare and to compete with other CFA. Besides the exact knowledge of upcoming training load, the workouts’ benchmark character together with their high status are also increasing intrinsic motivation when completing the workouts [13]. As CFA know their previous times and performance achievements, they might better plan targeted improvements based on lap times and appropriately selected timing. Finally, Hero-/Girl-WODs just reflect what CFA do in nearly every training session: workouts. Evidently, it is more likely to get better at something doing regularly (in this case workouts) than, for example, maximum strength performance that in turn is trained rarely [29].

This principle of increased performance by plural training repetitions also applies to the gymnastic elements. Despite being regular training exercises, they take up little time at low intensity, so that they can easily be practiced “in between” (e.g., hand stand hold or handstand walk). When practiced several times during set breaks or after WODs over a period of four weeks, those skills are also more likely to reach results faster compared to (maximum) strength. However, the most important aspect might be the following: The gymnastic elements comprise movements that are highly demanding in terms of coordination and motor skills. As already theoretically explained in detail, sleep plays a primary role in motor and cognitive learning and consolidation [32, 35]. Therefore, especially the technical movement sequences of gymnastic elements might be absorbed and consolidated during sleep [19, 27]. In contrast, sleep deprivation causes a reduced motor learning ability and an increased injury proneness, which in turn might explain the found differences between good and poor sleeping CFA [7]. Hence, in line with the assumptions from Kirschen and colleagues [22] and Watson [41], high sleep quality means an advantage of nightly processing of what has already been learned as well as an improved starting position to learn new movements and technical components efficiently and without injury.

However, as Olympic Weightlifting can also have a high due of technical and motor-related requirements, the question why no significant differences could be found within this CF discipline arises. Particularly, the assessment of maximum strength could be seen as critical. Measuring 1 RM correctly requires a certain know-how and targeted, structured training. On the one hand, maximum strength tests should be carried out within the mesocycle (lowering the volume in the previous week, so-called "tapering") and, on the other hand, be directly prepared on the respective assessment day (observing specific warm up sets, set breaks and timing, correct movement realization.). Furthermore, neither the three basic exercises (squat, deadlift, bench press) nor the dynamic exercises of Olympic Weightlifting (clean and jerk) are singular training disciplines in all CF programs. In many CF boxes, these strength exercises are not trained in a separate strength circuit, but rather within workouts. Thus, if no maximal strength training is carried out – even regardless of CFAs’ sleep quality – no major increases in maximum strength can be achieved.

To conclude, it should be noted that due to the nature of CF (i.e., various cognitive and motor skills required), not all performance categories can be compared equally with one another. Nevertheless, referring to the findings from Fullagar et al. [14] and Lastella et al. [25] the relationship between training load respectively performance outcome and athletes individual sleep behavior is often underestimated and might have much extensive impact. In line, the present study — as the first one dealing with sleep in the context of CF – showed that high sleep quality can be an important factor to consider in order to gain optimal performance outcomes in all different CF performance categories.

#### Other factors

Among other things, we asked for the rating of the effects of sleep quality on personal performance. Interestingly, beside the generally highly rated role of sleep quality, the evaluation of the two components “physical” and “mental” performance differed slightly. The influence of sleep quality on mental performance was rated higher than that on physical performance (see Fig. 2). Again, this reveals the positive psychological long-term effects of a healthy sleep behavior: In addition to better processing of emotions [3], that consciously as well as subconsciously mostly takes place during sleep, a good sleep hygiene and healthy sleep patterns lead to improved psychological well-being [4]. Likewise, Goel et al. [16] and Grandner [17] have summarized that the majority of people suffering from (chronic) sleep loss feel groggy, unfocused, unmotivated and aimless. On days with high sleepiness, many have problems passing their daily work/activities or tackle all upcoming tasks, including training sessions, with the needed motivation and concentration. Therefore, as we found that the sleep quality of CFA has an important influence on both, mental and physical performance, we provide the approaches of Bonnar and colleagues [1] and Halson [18] establishing tailored sleep monitoring methods and interventions, e.g., sleep hygiene strategies, to foster individual performance development in athletes.

#### Limitations

There are some constraints limiting our study: First, it is important to critically question the selection of the examination instrument. Although, due to the used online questionnaire, we were able to acquire a large and diverse group of participants, all results were only based on participants’ subjective self-assessment. Neither the actual sleep behavior nor the performance was quantified objectively. However, in line with well-reputed sleep research, it could be assumed that rather the sleep quality (i.e., subjective restoring and relaxation feelings) than the quantitative amount of sleep (i.e., total sleep time or number of awakenings) seems to be most essential for recovery and well-being. Whilst objective sleep assessment is restricted by environmental factors or instrument-related aspects, bias or gaps in memory and other personal characteristics are factors that might individually influence the answering of a questionnaire. Likewise, the quality of retrospectively collected data about participants’ performance development could be noted discussable. As there is no standardized CF performance testing framework/tool the methodological challenge was to sample the plural characteristics of CF and daily routines of CFA in feasible manner. Second, we examined the role of sleep on performance, i.e., considering performance-related outcomes as dependent variables. However, we neither considered the individuals’ need of recovery time respectively amount of sleep depending on their internal and external training load nor the fact that, in general, the ability to recover decreases with age and increases with increasing adaptation to increased training loads [39].

To conclude, data might be rather temporary than comprehensive due to various more or less controllable influencing factors. Hence, to achieve a holistic understanding of the relationship between sleep and CF performance we recommend mixed-methods designs for future studies.

## Conclusion and future directions

The current study reveals that high sleep quality can be a performance-enhancing factor in CF: The better CFA rated their individual sleep quality, the better were their performance outcomes. Especially in Hero-/Girls-WOD and Gymnastics, results were significant. However, future research is needed to gain a deeper insight in psychophysiological demands and sleep-related influences of performance as well as the daily training routine (periodization) in CFA.

In sum, the increasing awareness and optimization of CFA’ sleep behavior to maximize performance seems to be a promising approach. Finally, suitable sleep interventions to foster high sleep quality in CFA should be developed and empirically tested.

## Data Availability

The datasets used and/or analyzed during the current study are available from the corresponding author on reasonable request.

## Abbreviations

CF: CrossFit®
CFA: CrossFit® athletes
PSQI: Pittsburgh Sleep Quality Index
GS: Good sleepers
PS: Poor sleepers
